# Iridectomy combined with posterior approach anterior chamber gas injection technique: a novel technique for the treatment of extensive Descemet’s membrane detachment

**DOI:** 10.1186/s40662-025-00428-2

**Published:** 2025-03-09

**Authors:** Yu Shen, Yongqiao Chen, Fei Yin, Luyi Zhang, Xiaoxia Li, Jing Wu, Miaoqin Wu

**Affiliations:** 1 Rehabilitation Medicine Center, Department of Ophthalmology, Zhejiang Provincial People’s Hospital, Affiliated People’s Hospital, Hangzhou Medical College, Hangzhou, 310014 Zhejiang China; 2 Zhejiang Chinese Medical University, Hangzhou, 310053 Zhejiang China; 3 Hangzhou Medical College, Hangzhou, 310059 Zhejiang China

**Keywords:** Descemet’s membrane, Descemet’s membrane detachment, Iridectomy, Posterior approach anterior chamber gas injection

## Abstract

**Background:**

To present the iridectomy combined with posterior approach anterior chamber gas injection technique for the treatment of extensive Descemet’s membrane detachment (DMD), which is a novel surgical approach for the management of DMD after phacoemulsification.

**Case presentation:**

The surgical technique was performed on a 68-year-old female with a history of cataract phacoemulsification surgery and two times of anterior chamber gas injection to treat DMD. After creating a scleral tunnel at 4 o’clock of the limbus, the iris root in that direction was cut off. This was confirmed via an iris root incision indicating that the syringe needle entered the posterior chamber through the scleral tunnel. The anterior chamber was filled about 3/4 with 16% C3F8. After surgery, patients were required to maintain a supine position without pillows. One month post-surgery, the cornea was transparent, DMD had fully recovered, and the best corrected visual acuity improved to 20/20.

**Conclusions:**

The iridectomy combined with a posterior approach anterior chamber gas injection technique can be used as an alternative surgical option for the management of extensive DMD in patients who have undergone several ineffective anterior chamber gas injection surgeries.

## Background

Descemet’s membrane detachment (DMD) is a rare but serious complication after phacoemulsification surgery, which can lead to irreversible corneal decompensation, with an incidence rate of 0.044% to 0.5% [1–3]. Descemet’s-endothelial is responsible for maintaining the clarity of the cornea. The detachment of the Descemet’s membrane complex from the stroma leads to endothelial pump failure resulting in corneal stromal swelling, bullous keratopathy and significant loss of vision. Risk factors include blunt keratomes, excessive intraocular manipulation, an inadvertent injection of saline or viscoelastic material in the interlayer of the cornea, surgical eye with shallow chambers or hard nuclear cataracts, amongst others [1, 4–6].

Although spontaneous reattachment occurs in a very small number of patients with DMD, early treatment is crucial for visual rehabilitation, making surgical reattachment the preferred method for most patients. Descemetopexy using bubbles or isoexpansile gases has become the preferred treatment for simple DMD, while suturing can be used for the treatment of extensive DMD [7–10]. Moreover, once corneal endothelial decompensation occurs, endothelial keratoplasty (Descemet's stripping automated endothelial keratoplasty and Descemet’s membrane endothelial keratoplasty) can be considered as a treatment option [6]. Although the implementation of these measures may lead to the reduction of DMD, their operational complexity, significant injury risks, and potential complications hinder their widespread adoption.

Here, we propose a method combining iridectomy with posterior approach anterior chamber gas injection technique for the treatment of extensive detachment of the Descemet’s membrane in patients who have undergone several ineffective anterior chamber gas injection surgeries. To the best of our knowledge, this is a novel surgical approach for the management of DMD after phacoemulsification.

## Case presentation

All procedures conformed to the Declaration of Helsinki, and written informed consent was obtained from the participant. The patient is a 68-year-old female who underwent cataract phacoemulsification surgery with a 3.0 mm corneal incision on her left eye at another institution. After surgery, the patient’s best corrected visual acuity did not improve and the DMD was observed, so an injection of an air bubble into the anterior chamber was performed at 4 days after surgery. The DMD persisted for 1 week after treatment. Therefore, on the 12th day after cataract surgery, the patient was transferred to our hospital.

**Fig. 1:**
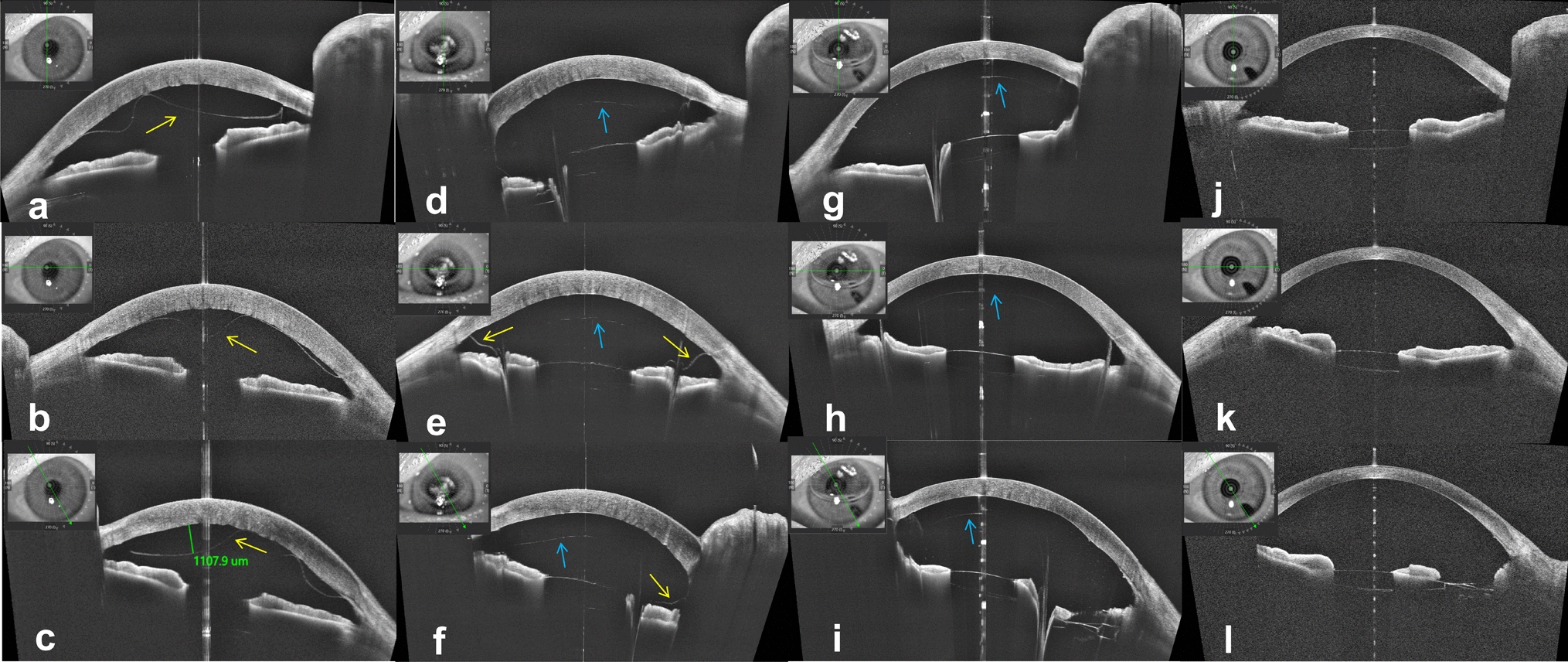
Morphological changes of Descemet’s membrane on AS-OCT. **a–c** AS-OCT showed extensive DMD throughout the cornea, without obvious scrolling. **d–f** AS-OCT showed that the DMD was aggravated and adhered to the surface of iris. **g–i** AS-OCT indicated the reattachment of Descemet’s membrane, accompanied by a slightly rough internal surface. **j–l** DMD had completely disappeared, and the cornea was transparent with no bubble in the anterior chamber. The yellow arrows represent the detached Descemet’s membrane, and the blue arrows represent the bubble. AS-OCT, anterior segment optical coherence tomography; DMD, Descemet’s membrane detachment

The best-corrected visual acuity was 20/2000, and the intraocular pressure in the left eye was 15 mmHg. Although diffuse corneal edema and DMD were observed on slit-lamp examination, the details were unclear. The anterior segment optical coherence tomography (AS-OCT) instrument used in this study was a swept-source OCT system (VG200D, SVision Imaging, Ltd., Luoyang, Henan, China). It showed extensive DMD throughout the cornea, with a maximum height of 1107.9 μm at the 11 o’clock position, but no obvious scrolling was observed (Fig. 1a–c).

Due to extensive DMD and severe visual impairment, the secondary anterior chamber gas injection was a convenient but necessary treatment option. After routinely disinfecting the patient’s left eye, paracentesis of anterior chamber was applied at 2 o’clock on the limbus of the cornea, 16% C3F8 was injected to fill the entire anterior chamber, and this was maintained for 10 min. Then, the gas was slowly released and we maintained a filling of about 3/4 of the anterior chamber with gas.

Five hours after the surgery, the patient suddenly complained of obvious pain in the operated eye. We hypothesized that the high intraocular pressure was caused by a pupillary block. Thus, a small amount of gas was released from the puncture site to alleviate the eye pain.

Although there was still 2/3 volume of gas in the anterior chamber on the second day of examination after surgery, diffuse corneal edema persisted. Due to the influence of bubbles, we observed aggravated DMD in the peripheral cornea on AS-OCT, which showed that it had adhered to the surface of iris. It indicated further aggravation of DMD (Fig. 1d–f). Therefore, we concluded that the injected gas did not successfully enter the anterior chamber. Instead, it entered between the corneal stromal layer and the Descemet’s membrane.

Repeated injection of gas was considered less likely to be successful, as due to extensive DMD, any puncture injection point at the corneal edge may cause gas to enter between the Descemet’s membrane and the stromal layer. Therefore, we considered other procedures for restoring Descemet’s membrane, such as suturing the Descemet’s membrane and endothelial transplantation, which appeared to be too complex and could cause too much damage to the cornea. Moreover, high intraocular pressure caused by pupil obstruction also presented as a problem. Hence, we proposed the combination of iridectomy with posterior approach anterior chamber gas injection technique for the treatment of extensive Descemet’s membrane detachment. The patient was informed of the surgical process and agreed to continue with the treatment.

**Fig. 2:**
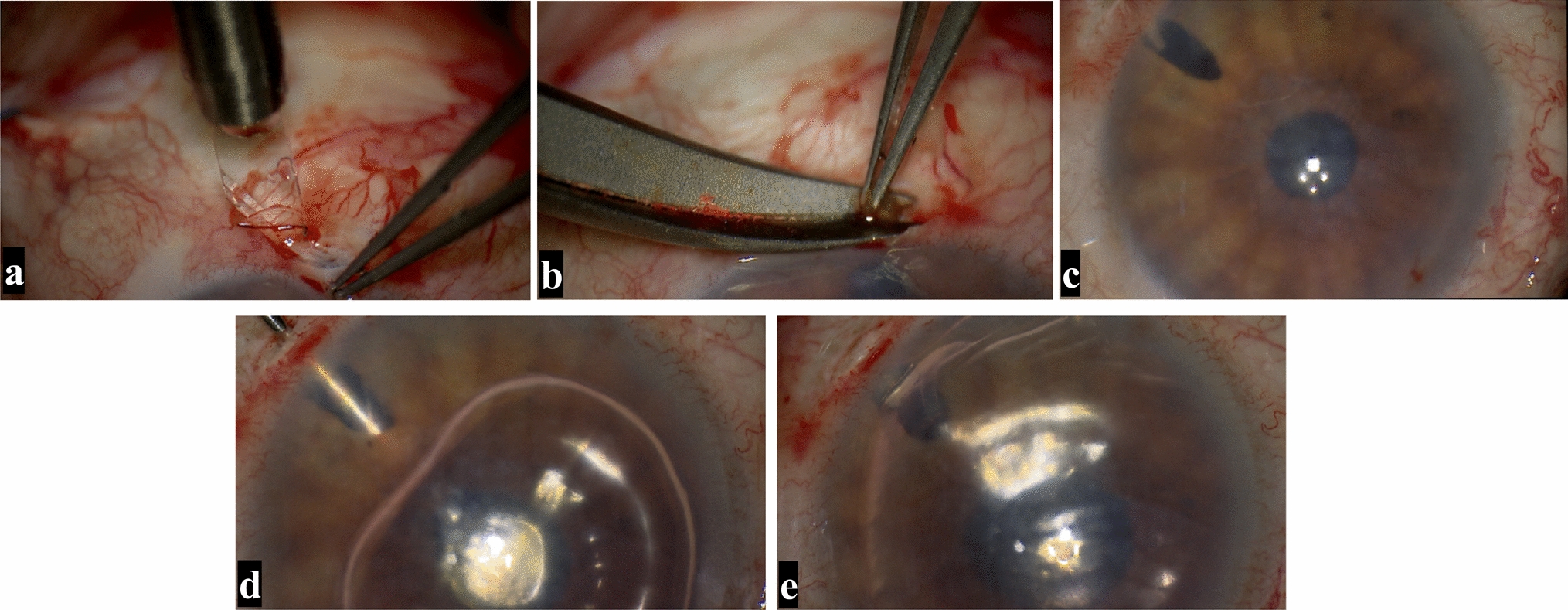
Surgical procedures performed in the iridectomy combined with posterior approach anterior chamber gas injection technique. **a** A scleral tunnel has been created at the 4 o'clock position of the limbus. **b** The iris root in that direction was cut off. **c** The pupil was constricted. **d** The syringe needle has entered the posterior chamber through the scleral tunnel and injected gas. **e** A bubble in the anterior chamber

**Fig. 3:**
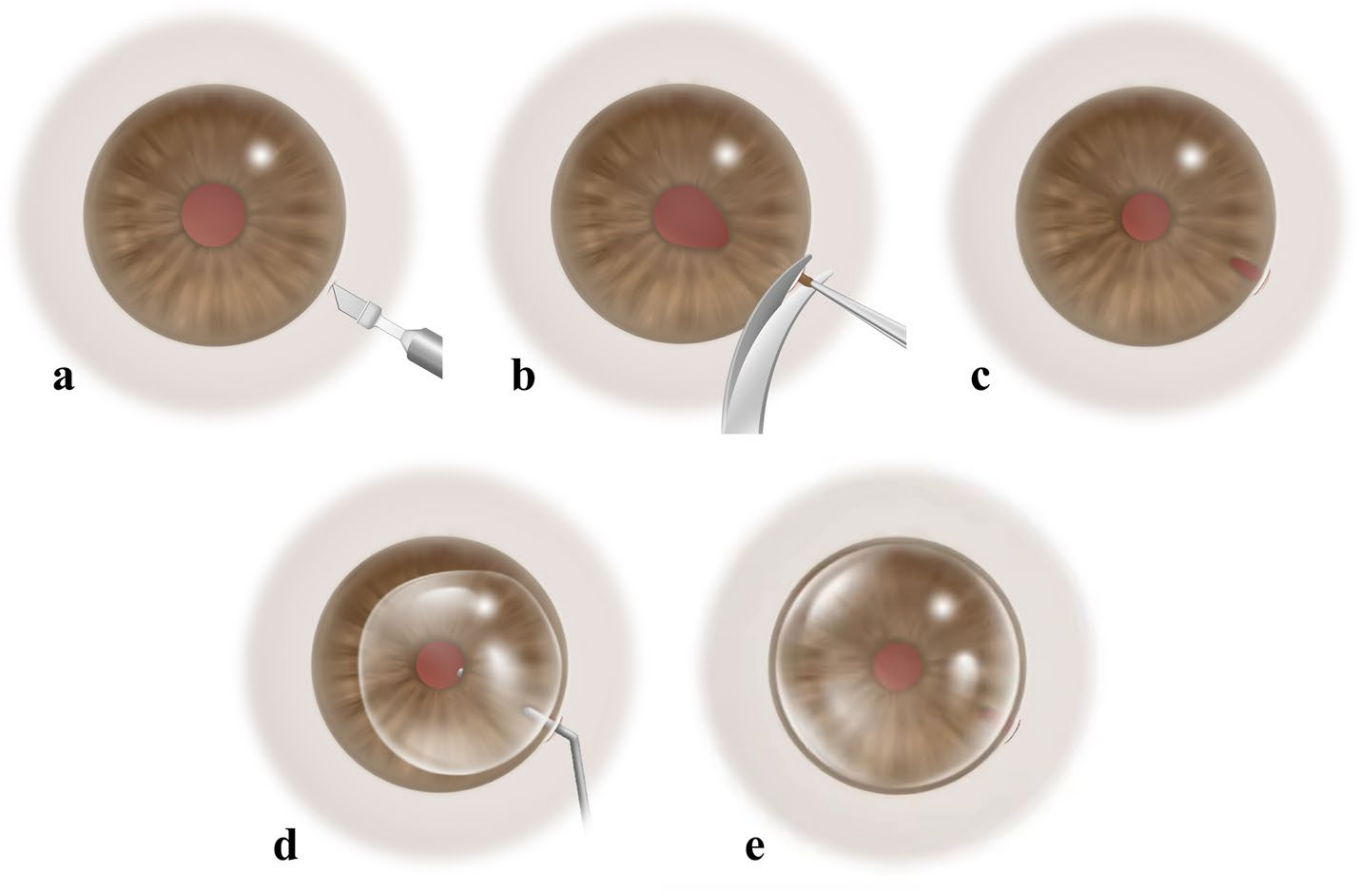
Diagrammatic illustration of the iridectomy combined with posterior approach anterior chamber gas injection technique. **a** Create a scleral tunnel at the inferior-temporal quadrant of the limbus. **b** Cut off the iris root in that direction. **c** Constrict the pupil. **d** The syringe needle enters the posterior chamber through the scleral tunnel and injects gas. **e** A bubble in the anterior chamber

For better eye immobilization and pain relief, the surgery was performed under retrobulbar anesthesia. After creating a scleral tunnel at 4 o’clock of the limbus, the iris root in that direction was cut off. Then, a puncture was performed at the termination point of the Descemet’s membrane at 11 o’clock to release the original gas, and the pupil was constricted with carbachol. This was confirmed via an iris root incision indicating that the syringe needle entered the posterior chamber through the scleral tunnel. The anterior chamber was filled about 3/4 with 16% C3F8. Figure 2 shows the surgical procedures performed, and Fig. 3 shows the diagrammatic illustration. After surgery, the patient was required to maintain a supine position without pillows, and antibiotics and corticosteroids were used locally to treat infection and inflammation.

On the first day after surgery, the intraocular pressure was normal. Under the slit lamp examination, corneal edema was relieved compared to before, with 2/3 of the anterior chamber filled with gas bubble and an iris root incision visible at the 4 o’clock position. The AS-OCT examination indicated the reattachment of Descemet’s membrane, accompanied by a slightly rough internal surface (Fig. 1g–i). On the third day after surgery, corneal edema subsided with significant improvement in corneal transparency, and 1/2 of the bubble remained in the anterior chamber, with vision improving to 20/333. One month after surgery, the cornea was transparent with no bubble in the anterior chamber, and the best corrected visual acuity improved to 20/20. DMD was found to have completely disappeared (Fig. 1j–l).

## Discussion and conclusion

Several mechanisms contribute to the occurrence of DMD following surgery. Firstly, mechanical factors during the surgical process can cause damage to the corneal structure. Secondly, intrinsic defects in the adhesion of the Descemet membrane result from functional defects in transforming growth factor beta 1 (TGF-β1). Additionally, other risk factors include a shallow anterior chamber and Fuchs’ corneal endothelial dystrophy [3, 11–13].

Currently, descemetopexy supported by anterior chamber gas injection is widely accepted, boasting a treatment success rate of 90% to 95% [1, 3, 14, 15]. Based on the extent of detachment, DMD is classified into large-area and small-area detachment. Detachment exceeding 1 mm is classified as large-scale detachment. Clinical signs such as Descemet’s membrane tears and rolled edges may indicate poor prognosis [11–13].

While the literature supports the use of 100% air or gases like C3F8 and SF6 for managing DMD, reports highlighting strategies following failed pneumodescemetopexy are few and far between. In long standing cases, transcorneal suturing has also been attempted to improve the chances of attachment [16], while for the impacted DMD, combined disimpaction of Descemet’s membrane and air descemetopexy can be an effective treatment option [17]. In this case report, the patient experienced DMD and first underwent an ineffective anterior chamber gas injection surgery at another institution. Subsequently, another anterior chamber gas injection surgery was performed at our hospital, which further worsened the DMD. We considered the presence of tears in the DM and rolled edges, which might be caused by multiple anterior chamber manipulations, leading to the entry of injected gas between the corneal stromal layer and the Descemet’s membrane, rather than inside the anterior chamber.

At present, there exists no definitive treatment for extensive DMD similar to the condition of our patient. Most groups support repeated anterior chamber gas injection, despite its lower likelihood of success. In cases of extensive DMD, any limbal puncture for gas injection may allow gas to track between Descemet’s membrane and the stromal layer, further complicating the detachment. Additionally, alternative interventions such as suturing the posterior elastic layer of the cornea or performing corneal endothelial transplantation are complex procedures that can cause significant corneal trauma [1, 7–10]. Moreover, it has been reported that a combination of intracameral gas injection and transscleral suturing can effectively repair DMD that does not reattach with the use of air alone. Keratoplasty should be considered as a last resort when all other treatments are proven ineffective [6]; though the risk of graft detachment after DMEK ranges from 2% to 82% [18]. Coco et al. suggested that the presence of stromal ripples was significantly correlated with the risk of graft detachment requiring retreatment, the risk of detachment of previously attached grafts, and the risk of worsening detachment over time. Furthermore, patients with mild or severe ripples exhibit a higher central corneal thickness compared to those without ripples [19]. Moreover, Lohmann et al. suggested that the likelihood of requiring rebubbling was extremely low in the absence of posterior stromal ripples, provided that graft detachment was less than one-third of the corneal area, central corneal thickness (CCT) had not decreased by more than 600 µm from preoperative values, and best-corrected visual acuity (BCVA) had improved within 1 week following uncomplicated DMEK [18]. We found that the situation was exacerbated by the occurrence of high intraocular pressure caused by pupillary block following the surgery. Therefore, we propose combining iridectomy with the posterior approach of anterior chamber gas injection for the treatment of extensive DMD.

During surgery, we created an iris root incision and infused gas from the posterior chamber into the anterior chamber through this incision under direct view. This prevented gas from entering the interface between Descemet’s membrane and the stromal layer, thereby mitigating the risk of DMD exacerbation and postoperative gas pupillary blockage. Considering previous phacoemulsification and failed gas injection on the superior limbus of cornea, it was more appropriate to make the iris root incision on the inferior limbus to reduce further eye damage. Moreover, the temporal space was relatively large and easy to operate. Therefore, we performed the peripheral iridectomy at the 4 o’clock position of the inferior-temporal quadrant. Constriction of the pupil was to make the incision on the iris root become visible quickly, so that the injection needle could more easily and accurately enter the posterior chamber through the scleral tunnel. It could also reduce the area of communication between the anterior and posterior chambers in the pupil, concentrate the force of injecting gas into the anterior chamber through the pupil, and facilitate reattachment of Descemet’s membrane. Additionally, we employed C3F8 for anterior chamber filling, a preferred choice for patients with complicated DMDs or failed DMDs. Some studies have indicated that for patients with extensive and severe DMD, transcorneal suturing or endothelial keratoplasty might be considered as treatment options, while these procedures are characterized by considerable difficulty, significant trauma, high costs, and numerous complications. Thus, our surgical approach addresses these limitations and achieves favorable outcomes in a short timeframe.

In conclusion, the iridectomy combined with posterior approach anterior chamber gas injection technique can be used as an alternative surgical option for the management of extensive Descemet’s membrane detachment patients who have undergone several ineffective anterior chamber gas injection surgeries.

## Data Availability

All data supporting our findings are provided within the manuscript.
